# Actinomycosis of the Forearm: An Unusual Presentation of an Early Event

**DOI:** 10.7759/cureus.69194

**Published:** 2024-09-11

**Authors:** Duarte Flor, Inês Coutinho, Francisca Morgado, Jose C Cardoso

**Affiliations:** 1 Department of Dermatology, Coimbra Local Health Unit, Coimbra, PRT

**Keywords:** actinomycosis, cutaneous bacterial infection, dermatopathology, immunosuppression, pustules

## Abstract

Actinomycosis is a rare chronic infectious disease, most commonly affecting the cervicofacial, pulmonary, or genitourinary areas. It is caused by the Actinomyces spp. bacteria, which are facultative anaerobes and gram-positive, nonsporing rods. The disease is* *characterized by the formation of cold abscesses or fistulas, as well as granulomatous tissue, reactive fibrosis, and necrosis, which can resemble local malignancy. Cutaneous actinomycosis in the trunk or extremities is extremely rare and is usually related to previous surgical procedures or trauma. However, we report one such rare case of cutaneous actinomycosis of the forearm without previous precipitating factors, which was successfully treated with prolonged antibiotherapy.

## Introduction

Actinomycosis is a rare, chronic, infectious disease caused by the anaerobic bacteria Actinomyces spp., which is commensal in the human gastrointestinal and genitourinary tracts. The most frequent presentations include cervicofacial actinomycosis, pulmonary actinomycosis, and genitourinary actinomycosis [1]. Actinomycosis presents with a protracted course, forming cold abscesses or fistulas, granulomatous tissue, reactive fibrosis, and necrosis, which may mimic local malignancy [2]. Bacterial cultures and pathology are essential to the diagnosis. The mainstay of treatment consists of prolonged high-dose antibiotherapy, with beta-lactams being the most frequently used [1,2].

Limb involvement in actinomycosis is extremely rare and usually associated with previous trauma or surgery. However, some cases have been described without a precipitating incident or relevant comorbidities [3,4]. This report presents an extremely rare case of cutaneous actinomycosis of the forearm.

## Case presentation

**Figure 1:**
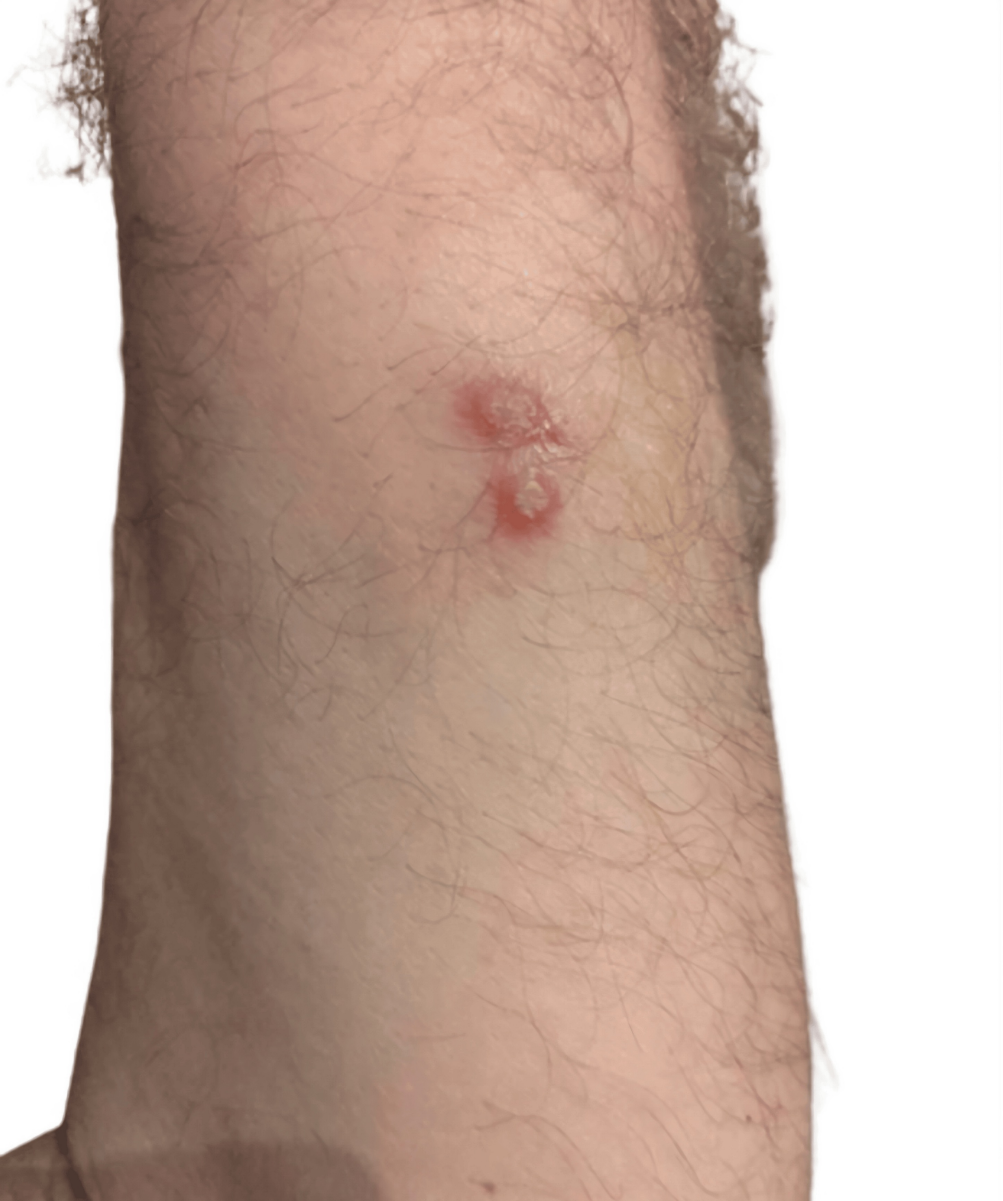
Initial presentation as photographed by the patient

A healthy 51-year-old male presented with asymptomatic cutaneous lesions on the left forearm that had been evolving for a month. On observation, two adjacent 5 mm wide pustules with an infiltrated erythematous base were visible, as shown in Figure 1, which details the morphology of the lesion's onset. He denied any local trauma or allergies.

He was treated with topical fusidic acid and betamethasone for two weeks with no improvement upon reobservation. One of the lesions developed superficial necrosis, and two new adjacent lesions appeared, prompting a biopsy.

**Figure 2:**
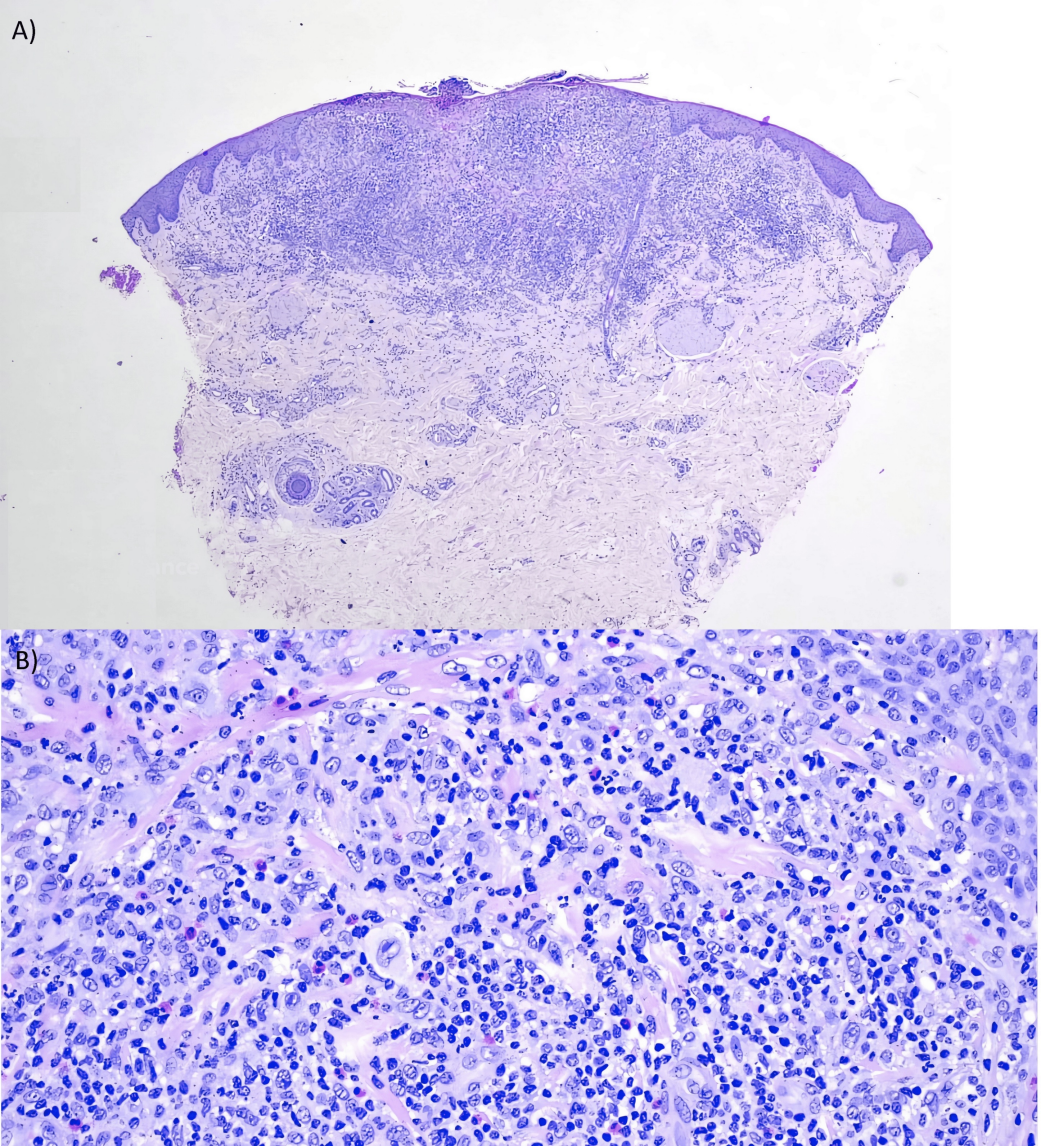
Epidermotropic dermal infiltrate of triangular morphology with inferior apex (A) containing lymphoid cells with indented nuclei and prominent nucleoli (B)

Pathology reported findings of a focal superficial crusted scale with an epidermotropic dermal infiltrate. The infiltrate had a triangular morphology with an inferior apex and contained small lymphocytes, large lymphoid cells with indented nuclei and prominent nucleoli, and histiocytic cells. Coexistent neutrophils and eosinophils were also observed (Figure 2). Immunohistochemistry showed predominant CD3-positive T cells, with numerous B cells in depth. CD30 highlighted numerous large lymphoid cells with a membranous pattern and paranuclear highlight. These cells coalesced into aggregates, which were identifiable in the dermal infiltrate and epidermis. The majority of cells were CD4-positive, outnumbering the CD8-positive cells. CD163 confirmed histiocyte presence.

**Figure 3:**
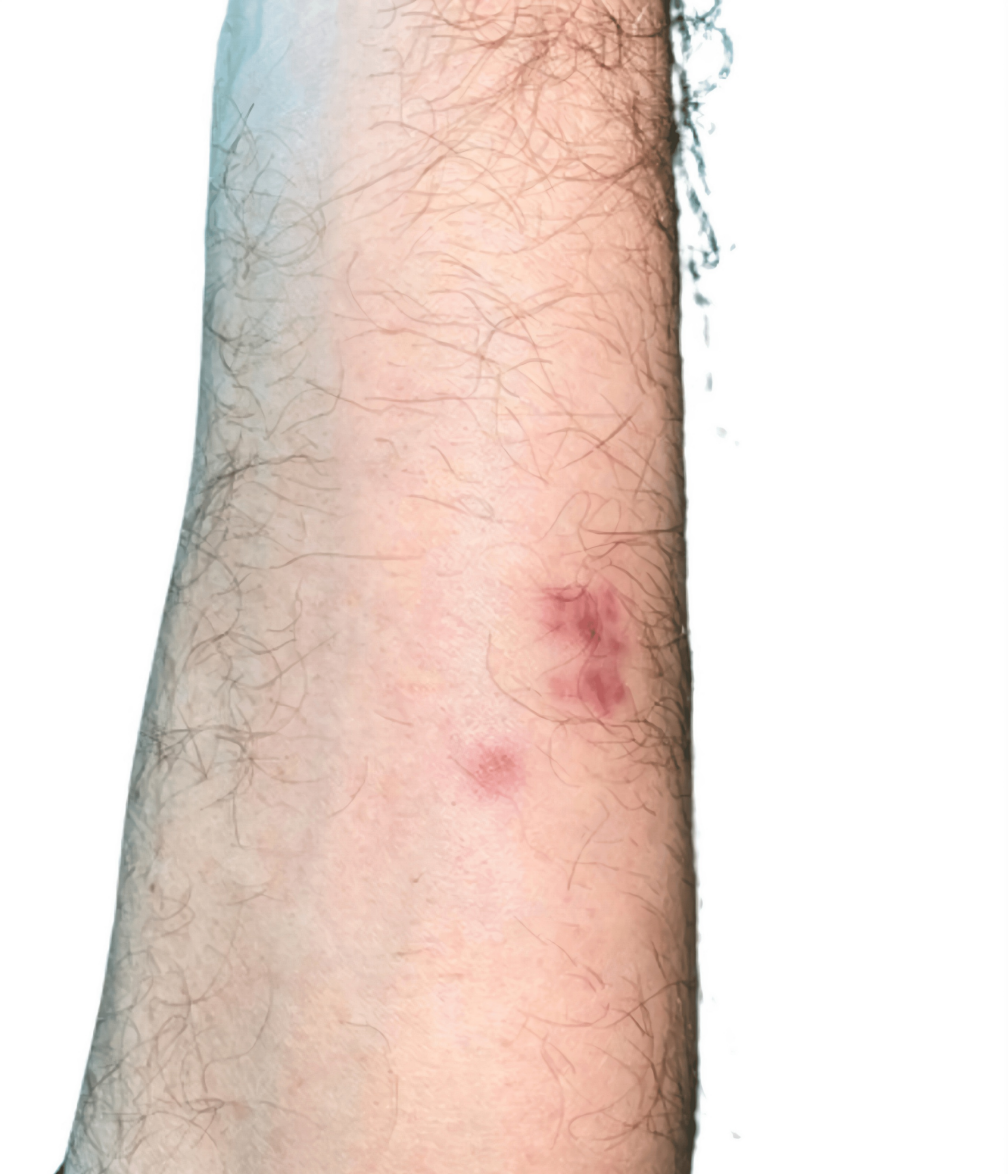
Resolution with postinflammatory hyperpigmentation

Cultures yielded positive results for *Actinomyces oris*, and the diagnosis of actinomycosis was therefore established. Oral amoxicillin was started. One month later, follow-up showed resolution of lesions, with only residual hyperpigmentation (Figure 3), and antibiotherapy was discontinued. Close monitoring was maintained for a year, with no recurrences. The analytical study ruled out relevant comorbidities or immunosuppression.

## Discussion

Actinomycosis is an uncommon, slowly progressive infection caused by the facultative anaerobe, gram-positive, nonsporing rod Actinomyces spp. (most frequently *Actinomyces israelii*). Infection typically develops in the context of local trauma or localized or systemic immunosuppression, with cervicofacial actinomycosis being associated with dental infections and pulmonary disease with smoking. Other locations, such as pharyngeal, gastric, genitourinary, and cutaneous actinomycosis, are often related to previous surgical procedures or trauma [1].

Clinical presentation varies according to the location and is unspecific. Cutaneous actinomycosis can present as subcutaneous nodular lesions slowly progressing into abscesses or fistulas with active draining [3-5]. Laboratory findings are unremarkable, as systemic involvement is typically absent, although hematogenic dissemination has been reported in other forms of actinomycosis [1].

Microbiological isolation of Actinomyces in skin specimens, preferably through surgical biopsy, can help with diagnosis. However, it occurs infrequently due to the extended culture time (at least 10 days) and the need for appropriate media. Additionally, concomitant infection or contamination by other organisms is possible. Gram stain is more sensitive, especially for patients under previous antibiotherapy [2].

Histological examination reveals a chronic granulomatous infection, often with tissue necrosis, characterized by the formation of sulfur granules with a surrounding rim of eosinophilic material (Splendore-Hoeppli phenomenon). These findings, coupled with the identification of filamentous, branching Gram-positive bacteria in the periphery of the granule, suggest the diagnosis [6].

The inherent complexities of microbiological and histological examinations make these cases susceptible to misdiagnosis owing to their nonspecific presentation. Nocardiosis, tuberculosis, and malignant disease are common differential diagnoses of actinomycosis.

Treatment of Actinomyces involves prolonged antibiotic therapy. The choice of the therapeutic agent, dose, and duration depends on the location and severity of the infection. Actinomyces spp. responds well to beta-lactams such as penicillin G or amoxicillin, and drug resistance is rare. These are usually the first choice. Macrolides and clindamycin can be used as alternatives [1-5]. Therapy is usually continued for months, and recurrences are not uncommon. Adjuvant surgical debridement has been described and may potentially decrease the necessary duration of antibiotic therapy [7]. Close follow-up to monitor for any potential recurrence is essential. This case of cutaneous actinomycosis is highly unusual due to its location and very early diagnosis, which may justify its atypical clinical and histological presentation.

## Conclusions

Cutaneous actinomycosis of the forearm is a rare clinical presentation of an uncommon infection, leading to its atypical clinical and histological presentation. Accurate diagnosis is essential for prompt and effective treatment.

Our case underlines the need to consider even atypical presentations of rare diseases in patients with odd clinical manifestations of disease. Histological and, when in doubt, microbiological examination often offers the solution for these difficult cases. In our case of confirmed forearm actinomycosis, resolution of all lesions ensued after one month of antibiotic treatment with no recurrences, although such quick resolution is not always the norm.
